# PP28 The Final Arguments To Incorporate Or Not Incorporate Technologies For Ultra-Rare Diseases Into The Brazilian Public Healthcare System

**DOI:** 10.1017/S0266462324002010

**Published:** 2025-01-07

**Authors:** Monica Aparecida de Paula de Sordi, Juliana Machado-Rugolo, Lehana Thabane, Marisa Santos, Denis Satoshi Komoda, Luis Gustavo Modelli de Andrade, Daniel da Silva Pereira Curado, Silke Anna Theresa Weber, Marilia Mastrocolla de Almeida Cardoso

## Abstract

**Introduction:**

The decision-making process for health technology assessment (HTA) in ultra-rare diseases is a global challenge. Establishing a comprehensive analytical framework for these unique diseases poses difficulties. This study aims to descriptively analyze arguments reported by the Brazilian National Committee for Health Technology Incorporation (Conitec) in deciding whether to include technology for ultra-rare diseases.

**Methods:**

Data from recommendation reports (2012 to 2022) were analyzed. Diseases with a prevalence of fewer than one per 50,000 inhabitants were classified as ultra-rare. Extracted variables included preliminary and final recommendation results and justifications by Conitec. Six argument categories were created (method-related issues; evidence; cost; technology effectiveness or safety; context; innovation). Word clouds were generated based on word frequency in each category to present the data.

**Results:**

In the analysis of 45 reports, the word clouds highlight frequent terms in favorable arguments, emphasizing evidence quality, cost reduction, and applicability in the healthcare system. Conversely, unfavorable arguments also revolve around evidence quality and cost impact. The analysis of the arguments according to categories, 16 arguments were identified: seven concern evidence issues, five cite methodological problems in presented studies, four relate to costs, and three pertain to technology effectiveness or safety. Unfavorable arguments primarily stem from evidence-related concerns. In favorable arguments, cost (seven) and safety (six) are prominent, with innovation (one) and context (three) being additional categories not found in the unfavorable group.

**Conclusions:**

While technology assessment processes for ultra-rare diseases have evolved, the justifications for recommending or not incorporating new technologies remain unchanged. Over time, reports have become more detailed, focusing on evidence and methodological specifics. This highlights the importance of scrutinizing evidence characteristics and determining relevant criteria and data types for this unique context.

